# The study of effect of didecyl dimethyl ammonium bromide on bacterial and viral decontamination for biosecurity in the animal farm

**DOI:** 10.14202/vetworld.2018.706-711

**Published:** 2018-05-27

**Authors:** Tippawan Jantafong, Sakchai Ruenphet, Darsaniya Punyadarsaniya, Kazuaki Takehara

**Affiliations:** 1 Department of Immunology and Virology, Faculty of Veterinary Medicine, Mahanakorn University of Technology, Bangkok, Thailand; 2 Laboratory of Animal Health, Department of Veterinary Medicine, Faculty of Agriculture, Tokyo University of Agriculture and Technology, Japan

**Keywords:** bactericidal, didecyl dimethyl ammonium bromide, disinfectant, quaternary ammonium compound, virucidal

## Abstract

**Aim:**

The aim of this study was to determine the effectiveness of the fourth-generation quaternary ammonium compounds, didecyl dimethyl ammonium bromide (DDAB), on the efficacy of bacterial and viral decontamination against pathogens commonly found in livestock industry including *Salmonella* infantis (SI), *Escherichia coli*, and avian influenza virus (AIV).

**Materials and Methods:**

The DDAB was prepared at 500, 250, and 125 parts per million (ppm) for absent and present organic material. Meanwhile, 5% of fetal bovine serum in DDAB solution sample was used to mimic the presence of organic material contamination. 400 µl of each DDAB concentration was mixed with 100 µl of each pathogen (SI, *E. coli*, and AIV) and then incubated at room temperature or 4°C at various time points (5 s, 30 s, 1 min, 5 min, 10 min, 15 min, and 30 min). The activity of DDAB treatment was stopped using 500 µl of FBS. Each treatment sample was titrated on either deoxycholate hydrogen sulfide lactose agar plates or Madin-Darby canine kidney cells for bacteria and AIV, respectively. Each treatment was conducted in triplicates, and the pathogen inactivation was considered effective when the reduction factor was ≥3 log_10_.

**Results:**

Our current study revealed that the DDAB inactivated SI, *E. coli*, and AIV under the various concentrations of DDAB, organic material conditions, exposure temperature, and exposure timing. In addition, the comparison of bactericidal and virucidal efficacy indicated that bacteria were more susceptible to be inactivated by DDAB as compared to viruses. However, DDAB showed marked inactivated differences in the absence or presence of organic materials.

**Conclusions:**

The DDAB may be a potential disinfectant for inactivating bacteria and viruses, especially enveloped viruses, in livestock farms. It can be useful as a disinfectant for biosecurity enhancement on and around animal farm.

## Introduction

Animal food products such as eggs, meat, and milk are considered as vehicles for one or more of pathogens causing foodborne illnesses [1] such as *Escherichia coli*, *Salmonella* spp., and Avian influenza virus (AIV) and for zoonotic disease [2]. Several serovars of *Salmonella* regularly found in food sources, especially poultry products, including eggs and poultry meat can cause detrimental salmonellosis in human. *Salmonella* is the main causative agent of foodborne disease outbreaks, which led to the economic loss of $3.7 billion per year globally, with severe impacts in public health [3]. Giraudon *et al*. [4] demonstrated that inadequate disinfection of equipment and surfaces is a critical attribute for the outbreak of *Salmonella*. *E* *coli* is the most common bacteria causing disease in animals, especially the poor management and sanitation, leading to the high occurrence of that disease [2]. In the past decade, AIV has become one of the dominant zoonotic pathogens with severe impacts in the poultry industry. The combination of the use of vaccine, disease surveillance, and strict biosecurity has been demonstrated to be a most effective measure for AIV control [5]. In Thailand, these three pathogens have been major diseases causing organisms with high morbidity in livestock, particularly in poultry farms [6-8].

Biosecurity is one of the most potent strategies for disease prevention and control, especially against zoonotic infections. As proper disinfection in farm can enhance disease prevention and control, the potency of disinfectant is essential to ensure effective decontamination of livestock farm. Disinfectants are routinely used to sanitize equipment, tools, floors, walls, and vehicles among others [9]. There are various types of disinfectant used in animal farms such as alcohols, aldehydes, halogens, phenols, oxidizing agents, and quaternary ammonium compounds (QACs). Didecyl dimethyl ammonium bromide (DDAB) is the fourth generation of QACs comprising of numerous products that are widely used for their antimicrobial properties and are applied in industrial products and household [10]. In Thailand, QACs products are extensively used in animal farms; however, the study on the efficacy of DDAB against certain organisms including *Salmonella* infantis (SI), *E. coli*, and AIV remains unexplored.

The aim of this study was to evaluate the efficacy of DDAB against SI*, E. coli*, and AIV for farm biosecurity under various mimic natural conditions such as the absence or presence of organic materials, varying exposure temperature, and time.

## Materials and Methods

### Ethical approval

Ethical approval is not applicable to this study due to no animal testing.

### Disinfectant preparation

The DDAB (Bestaquam-S^®^, China Bestar Laboratories Ltd., Taiwan) was diluted as 500, 250, and 125 part per million (ppm) in sterile distilled water for absent and present organic material. Five percent of fetal bovine serum (FBS) in DDAB solution sample was used to mimic the presence of organic material contamination. In the presence of 5% organic material, 500 μl of FBS was added to 10 ml of each DDAB solution sample (500, 250, and 125 ppm) before testing.

### Pathogens, cell culture, and medium

The *E. coli* and SI were used as representative pathogenic bacteria in this study. Bacteria were cultured on selective medium, namely, deoxycholate hydrogen sulfide lactose (DHL) agar and incubated into 37°C incubator overnight. For working bacteria preparation, a single bacterial colony was picked and cultured in Luria-Bertani medium (1% Bacto Tryptone, 0.5% Bacto Yeast Extract, and 1% NaCl, pH 7.4) following the method previously described by Ota *et al*. [11]. Before microbiological testing, both bacterial cultures were centrifuged at 1750× *g* for 10 min to remove organic materials.

The low pathogenic AIV, namely, A/duck/Aomori/Japan/395/2004 H7N1 was propagated in 9-day-old chicken embryonic eggs. At 3-day post-inoculation, allantoic fluid was harvested and kept at −80°C until testing. The Madin-Darby canine kidney (MDCK) cells were used for AIV titration.

### Blocking solution preparation

DDAB activity was attenuated by blocking solution using 100% of FBS in an equal volume of sample solution and pathogen.

### Bactericidal and virucidal testing

This experiment was performed according to the method of Sonthipet *et al*. [12]. Briefly, 400 µl of each DDAB solution sample was mixed with 100 µl of SI, *E. coli*, or AIV and incubated at room temperature of 4°C for various exposure/contact times such as 5 s, 30 s, 1 min, 3 min, 5 min, 10 min, 15 min, or 30 min. The activity of DDAB treatment was stopped using 500 µl of FBS, and then, bacteria or virus was titrated onto DHL agar plates or MDCK, respectively. The neutralizing efficacy of blocking solution was confirmed by adding the FBS into each DDAB sample before virus or bacteria inoculation, namely, 0 s. All treatment was prepared in triplicates for examination, and the mean titers were generated with standard deviation.

### Virus and bacteria titration and calculation

This experiment was performed the following previous study by Sonthipet *et al*. [12]. Briefly, each *E. coli* and SI treatment was diluted as 10-fold serial dilution using phosphate buffer saline and titrated onto DHL agar Petri dishes for bacterial titration. All titrating Petri dishes were incubated in a 37°C incubator, and the number of colony was counted at 24 h post culturing. The bacteria titer was calculated in colony-forming units (CFU)/ml.

For AIV, this experiment was performed the following previous study by Thammakarn *et al*. [13]. The treated sample was diluted as 10-fold serial dilution using Eagle’s minimum essential medium (Nissui Pharmaceutical Co., Ltd., Tokyo, Japan) containing final concentration of 0.2 µg/ml of trypsin (Sigma, St. Louis, MO, USA). Each dilution was inoculated onto MDCK monolayer and incubated at 37°C in a 5% CO_2_ incubator. The cytopathic effect was observed twice a day for 3 days, and the hemagglutination test was conducted using the inoculating supernatant to confirm the presence of the virus. Finally, virus titration was determined using 50% tissue culture infective dose (ml) by Behrens and Kärber’s method.

### Inactivation analysis

To evaluate the bacterial or viral inactivation, the effective inactivation was analyzed using the reduction factor (RF). The RF equation is as follows: RF=t_pc_−t_a_, where t_pc_ is the titer of positive control, and t_a_ is the titer from treated sample [14,15]. The RF was ≥3 log_10_, indicating that bacterial or viral inactivation was effective.

## Results

### Bactericidal efficacy of DDAB

The bactericidal efficacy of DDAB against SI and *E. coli* in the absence or presence of organic materials at room temperature is shown in Tables-1 and 2, respectively. In the absence of organic materials, SI was inactivated within 5 s regardless of the concentration of DDAB. However, the effectiveness of DDAB was reduced in the presence of 5% organic material. DDAB at a concentration of 500, 250, and 125 ppm inactivation took place within 5 s, 1 min, and 1 min, respectively.

**Table-1:** The bactericidal efficacy of DDAB against SI in the absence or presence of organic materials.

Contact times	Absence organic materials			Presence organic materials^a)^		
	
	500	250	125	500	250	125
t_pc_^b)^	8.13±0.12	8.30±0.28	7.86±0.87	8.13±0.12	8.30±0.28	7.86±0.87
0 s^c)^	7.65±0.33	8.18±0.20	7.89±0.46	8.20±0.06	7.73±0.55	7.82±0.15
5 s^d)^	3.30±0.70*	4.30±1.01*	4.55±1.80*	4.52±1.14*	6.05±0.94	NT
30 s	<2.60±0.00*	3.88±1.11*	3.76±2.01*	3.27±1.15*	5.51±0.82	5.56±0.42
1 min	NT^e)^	3.41±1.39*	3.70±1.91*	3.25±1.13*	3.30±0.60*	4.47±1.03*
5 min	NT	NT	NT	NT	2.70±0.17*	3.57±1.68*
10 min	NT	NT	NT	NT	NT	3.94±2.31*
15 min	NT	NT	NT	NT	NT	<2.60±0.00*

The results are presented as log_10_ CFU/ml (mean±SD) of SI inactivating activity at room temperature using DDAB as 500, 250, and 125 ppm in the absence or presence of organic materials.

a) Fetal bovine serum was added to DDAB solution as 5% organic materials of total volume.

b) The titer converted into an index in log_10_ of bacteria control.

c) Added blocking solution before SI.

d) The titer converted into an index in log_10_ of the recovered bacteria after indicated time of treatment such as 5 s, 30 s, 1 min, 5 min, 10 min, and 15 min.

e) NT=Not tested,

* Inactivation effective when RF was ≥3. DDAB=Didecyl dimethyl ammonium bromide, SI=*Salmonella infantis*, CFU=Colony-forming units, SD=Standard deviation, RF=Reduction factor

**Table-2:** The bactericidal efficacy of DDAB against *E. coli* in the absence or presence of organic materials.

Contact times	Absence of organic materials			Presence of organic materials^a)^		
	
	500	250	125	500	250	125
t_pc_^b)^	8.61±0.36	8.61±0.36	8.61±0.36	8.61±0.36	8.61±0.36	8.61±0.36
0 s^c)^	7.19±0.43	8.01±0.78	8.34±0.46	7.77±0.67	8.30±0.12	8.42±0.20
5 s^d)^	<2.60±0.00*	<2.60±0.00*	4.73±1.33*	4.82±0.38*	5.40±0.35*	7.06±0.60
30 s	3.00±0.70*	<2.60±0.00*	3.19±1.03*	2.94±0.58*	4.45±0.65*	5.30±0.17*
1 min	NT^e)^	NT	3.35±0.84*	NT	2.86±0.45*	3.57±0.95*
5 min	NT	NT	3.04±0.75*	NT	NT	2.80±0.35*
10 min	NT	NT	NT	NT	NT	NT
15 min	NT	NT	NT	NT	NT	NT

The results are presented as log_10_ CFU/ml (mean±SD) of *E. coli* inactivating activity at room temperature using DDAB as 500, 250, and 125 ppm in the absence or presence of organic materials.

a) Fetal bovine serum was added to DDAB solution as 5% organic materials of total volume.

b) The titer converted into an index in log_10_ of bacteria control.

c) Added blocking solution before *E. coli*.

d) The titer converted into an index in log_10_ of the recovered bacteria after indicated time of treatment such as 5 s, 30 s, 1 min, 5 min, 10 min, and 15 min.

e) NT=Not tested,

* Inactivation effective when RF was ≥3. DDAB=Didecyl dimethyl ammonium bromide, CFU=Colony-forming units, SD=Standard deviation, RF=Reduction factor, *E. coli=Escherichia col*i

The result of *E. coli* inactivation is shown in Table-2. At 500, 250, and 125 ppm without organic materials contamination, took place within 5 s, 5 s, and 5 s, respectively. Even in the presence of organic material, inactivation took place within 5 s, 5 s, and for a longer period (30 s), respectively.

### Virucidal efficacy of DDAB

The virucidal efficacy of DDAB at a concentration of 500, 250, and 125 ppm against AIV in the absence or presence of organic materials at room temperature is shown in Table-3. 500, 250, and 125 ppm of DDAB inactivated AIV even in the absence of organic material within 5 s, 1 min, and 10 min, respectively. In the case of presence of 5% organic materials, inactivation occurred at 500 ppm within 15 min; however, at 250 and 125 ppm, no inactivation happened within 15 min (Table-3).

**Table-3:** The virucidal efficacy against AIV at room temperature.

Contact times	Absence of organic material			Presence of organic materials^a)^		
	
	500	250	125	500	250	125
t_pc_^b)^	7.25±0.43	7.08±0.38	7.67±0.63	7.25±0.43	7.08±0.38	7.67±0.63
0 s^c)^	6.75±0.25	7.25±0.43	7.33±0.29	6.75±0.90	6.92±0.58	7.50±0.66
5 s^d)^	2.67±0.29*	NT	NT	NT	NT	NT
30 s	<2.50±0.00*	4.92±0.88	NT	NT	NT	NT
1 min	NT^e)^	3.58±0.95*	6.88±0.18	NT	NT	NT
5 min	NT	<2.50±0.00*	5.75±0.35	NT	NT	NT
10 min	NT	NT	4.50±1.09*	4.92±1.04	6.42±0.29	NT
15 min	NT	NT	NT	3.92±1.66*	6.17±0.38	6.92±0.38
30 min	NT	NT	NT	2.67±0.29*	5.58±0.76	7.17±1.01

The results are presented as log_10_ TCID_50_/ml (mean±SD) of AIV inactivating activity at room temperature using DDAB as 500, 250, and 125 ppm in the absence or presence of organic materials.

a) Fetal bovine serum was added to DDAB solution as 5% organic materials of total volume.

b) The titer converted into an index in log_10_ of virus control.

c) Added blocking solution before AIV.

d) The titer converted into an index in log_10_ of the recovered virus after indicated time of treatment such as 5 s, 30 s, 1 min, 5 min, 10 min, 15 min, and 30 min.

e) NT=Not tested,

* Inactivation effective when RF was ≥3. DDAB=Didecyl dimethyl ammonium bromide, CFU=Colony-forming units, SD=Standard deviation, RF=Reduction factor, AIV=Avian influenza virus, TCID_50_=50% tissue culture infective dose

The virucidal efficacy of DDAB against AIV in the absence or presence of organic materials at 4°C is shown in Table-4. These results revealed that only 500 ppm of DDAB could inactivate AIV within 30 min in the absence of organic material. However, at 250 ppm without organic material, 500 and 250 ppm containing 5% of organic materials could not inactivate AIV within 30 min (Table-4).

**Table-4:** The virucidal efficacy against AIV at 4°C.

Contact times	Absence of organic material		Presence of organic materials^a)^	
	
	500	250	500	250
t_pc_^b)^	7.75±0.53	7.50±0.66	7.67±0.63	7.50±0.66
0 s^c)^	7.42±0.14	7.58±0.76	7.50±0.90	8.00±0.66
5 s^d)^	NT^e)^	NT	NT	NT
30 s	NT	NT	NT	NT
1 min	NT	NT	NT	NT
5 min	NT	NT	NT	NT
10 min	5.38±0.18	7.25±0.71	NT	NT
15 min	5.17±0.58	6.92±0.88	7.50±0.43	7.75±0.25
30 min	4.17±1.76*	6.83±0.52	7.33±0.76	7.50±0.75

The results are presented as log_10_ TCID_50_/ml (mean±SD) of AIV inactivating activity at 4°C using DDAB as 500 and 250 ppm in the absence or presence of organic materials.

a) Fetal bovine serum was added to DDAB solution as 5% organic materials of total volume.

b) The titer converted into an index in log_10_ of virus control.

c) Added blocking solution before AIV.

d) The titer converted into an index in log_10_ of the recovered virus after indicated time of treatment such as 5 s, 30 s, 1 min, 5 min, 10 min, 15 min, and 30 min.

e) NT=Not tested,

* Inactivation effective when RF was ≥3. DDAB=Didecyl dimethyl ammonium bromide, CFU=Colony-forming units, SD=Standard deviation, RF=Reduction factor, AIV=Avian influenza virus, TCID_50_=50% tissue culture infective dose

## Discussion

Nowadays, most Thai livestock farms have implemented good agricultural practice and livestock farm standards to ensure product safety and improve animal health [8]. The biosecurity measures have become a great strategy for disease prevention and control to minimize pathogen outbreak in animal farms. Disinfectants are a powerful tool in a biosecurity program [16]. In addition, appropriate selection of disinfectants is critical in establishing a successful biosecurity program. However, several attributes affect the efficacy of disinfectants such as the characteristic of pathogens, temperature, contact time, and organic load [17]. There are numerous disinfectants commercially available on livestock farms. QACs is one of the most disinfectants commonly used in Thailand [18]. QACs is used as biocides to eradicate a wide range of bacterial strains, especially, Gram-negative bacteria [19].

QACs was shown to be effective disinfectant against PCV2 and Equine influenza A virus [20,21]. In Thailand, after the last outbreaks of highly pathogenic avian influenza subtype H5N1 in 2008 [22], regular surveillance and strict biosecurity have become a great strategy for disease prevention in Thai livestock and poultry farms. The study by Wanaratana *et al*. [23] showed that combination of QACs with various disinfectant including chlorine, phenol, and glutaraldehyde worked synergistically to inactivate AIV subtype H5N1. In addition, a study by Krangvichain *et al*. [24] demonstrated that QACs was effective to eradicate opportunistic pathogenic yeast, *Cryptococcus neoformans* in fecal droppings from pigeons. In our study, we demonstrated the efficacy of QACs on decontamination of bacteria and viruses commonly found in animal farms including *Salmonella*, *E. coli*, and AIV for the first time.

The efficacy of blocking solution, namely FBS, is an instrument for exposure timing determination. The present study demonstrated that the titer of bacteria/virus control (t_pc_) compared with 0 s did not show marked inactivation difference, and these results indicated that this blocking solution could neutralize the inactivation activity of DDAB and utilize as instrument for determination of exposure or contact time for present study. The pathogens including SI, *E. coli*, and AIV, which represent serious microbial pathogens in Thai livestock, were susceptible to DDAB. However, the bactericidal and virucidal efficacies of DDAB were reduced in the presence of organic materials. This finding is consistent with the previous studies conducted in *Salmonella enterica* serovar Typhimurium and *Vaccinia virus* [16,25]. A study by Jang *et al*. [17] indicated that low temperature reduced the efficiency of QACs against *Salmonella enterica* serovar Typhimurium. A similar observation was observed in our study with regard to the virucidal efficacy of DDAB which greatly declined at 4°C. In our experiment, we found that bacteria are more susceptible to DDAB as compared to viruses. Disinfectants had differing product efficacy when applied to be used in the farm for various reasons; hence, it is necessary to establish the appropriate temperature, contact time, and optimum concentration for the best performance of this disinfectant.

## Conclusion

Several attributes including disinfectant concentration, temperature, contact time, and contaminated organic material account for the maximal efficiency of disinfectants; therefore, these attributes should be used as criteria for appropriate selection of disinfectants. We have shown that, at room temperature, DDAB at 125 ppm was effective to inactivate Gram-negative bacteria (SI and *E. coli*) within 5 s; however, in AIV, inactivation at a higher concentration at 500 ppm was required to achieve similar efficacy. The efficacy of DDAB was still effective even in the presence of organic materials, indicating that contaminated organic materials commonly found in environment minimally impact the efficacy of DDAB; however, greater efficacy will be observed when organic contaminants are first removed. We observed that inactivation capacity of DDAB greatly declined at 4°C as compared with at room temperature. This indicated that DDAB might not be very effective in the winter season, especially at the Northern and Southern Hemisphere countries. In this study, we have provided general guidelines including appropriate temperature, contact time, and optimal concentration that optimize the effectiveness of DDAB for the use in livestock farms. In conclusion, DDAB can inactivate bacteria and viruses, especially in the absence of organic material, and can be useful as a disinfectant for biosecurity enhancement on and around the animal farm.

## Author’s Contributions

KT supervised the study. TJ and SR designed and coordinated the study. TJ, SR, DP, and KT performed the experiment. TJ and SR analyzed the data and wrote the manuscript. The final manuscript has been read and developed in consultation with all authors.
